# Capillary Isoelectric Focusing Immunoassay for Fat Cell Differentiation Proteomics

**DOI:** 10.1371/journal.pone.0132105

**Published:** 2015-07-01

**Authors:** Mary G. Johlfs, Priyatham Gorjala, Yasuyo Urasaki, Thuc T. Le, Ronald R. Fiscus

**Affiliations:** Department of Biomedical Sciences, Center for Diabetes and Obesity Research, College of Medicine, Roseman University of Health Sciences, 10530 Discovery Drive, Las Vegas, Nevada, 89135, United States of America; University of Pecs Medical School, HUNGARY

## Abstract

Profiling cellular proteome is critical to understanding signal integration during cell fate determination. In this study, the capability of capillary isoelectric focusing (cIEF) immunoassays to detect post-translational modifications (PTM) of protein isoforms is demonstrated. cIEF immunoassays exhibit protein detection sensitivity at up to 5 orders of magnitude higher than traditional methods. This detection ultra-sensitivity permits proteomic profiling of several nanograms of tissue samples. cIEF immunoassays are employed to simultaneously profile three protein kinases during fat cell differentiation: cGMP-dependent protein kinase type I (PKG-I) of the nitric oxide (NO) signaling pathway, protein kinase B (Akt) of the insulin signaling pathway, and extracellular signal-regulated kinase (ERK) of the mitogen-activated protein kinase (MAPK) signaling pathway. Interestingly, a switch in the expression level of PKG- isoforms is observed during fat cell differentiation. While both PKG-Iα and PKG-Iβ isoforms are present in preadipocytes, only PKG-Iβ isoform is expressed in adipocytes. On the other hand, the phosphorylation level increases for Akt while decreases for ERK1 and ERK2 following the maturation of preadipocytes into adipocytes. Taken together, cIEF immunoassay provides a highly sensitive means to study fat cell differentiation proteomics. cIEF immunoassay should be a powerful proteomics tool to study complex protein signal integration in biological systems.

## Introduction

Proteins are highly dynamic macromolecules whose structures and functions vary as a function of post-translational modifications (PTM) [1]. PTM provide a robust means to increase functional diversity of the proteome [2]. PTM dynamically regulate all functional aspects of living cells [3]. Abnormal PTM of critical proteins are commonly associated with the causes or consequences of diseases [4–6]. PTM and post-translational proteolytic events also regulate cell signaling cascade [7], behavioral response [8], and fate determination [9]. Profiling cellular proteome as a function of health and disease is critical to understand disease pathogenesis and signal integration in biological systems [10].

Proteome profiling technologies have been evolving and advancing rapidly over the last several decades. High-throughput methods such as protein microarrays [11] or microfluidic lab-on-a-chip platforms [12] permit massively parallel analysis of thousands of parameters within a single experiment. However, these high-throughput methods are still in development and have not been accessible to most biological laboratories. On the other hand, two-dimensional gel electrophoresis is a highly accessible method for protein analysis. In 2D gel electrophoresis, proteins are separated first by charges with isoelectric focusing (IEF), then by molecular masss with SDS-polyacrylamide gel electrophoresis [13]. PTM generally cause changes in the protein isoelectric points and proteolytic cleavages lead to changes in protein molecular masss. Mass spectrometry analysis of proteins isolated from 2D gel is becoming a versatile proteomics tool [14,15]. Western blots following 2D gel electrophoresis (2D WB) remain the most widely used method for proteome profiling [16]. A drawback of 2D WB is the requirement of relatively high quantity of total cellular protein for analysis. This requirement hinders the application of 2D WB for clinical disease proteomics due to limited quantity of tissue biopsies.

An alternative to gel IEF is capillary IEF (cIEF) [17,18]. cIEF immunoassay provides a highly sensitive means to evaluate protein expression level and PTM. Commercial cIEF systems are now readily available for biological research laboratories with walk-away automation that increases sample throughput [19]. Using commercial cIEF systems, detection of picograms of recombinant proteins or nanograms of total protein lysate have been reported [19–22]. cIEF is well-suited for clinical disease proteomics because protein isoforms and PTM could be detected in tissue biopsies [23–25].

In this study, we describe the use of cIEF immunoassay to detect isoforms of protein kinase G type I, PKG-Iα and PKG-Iβ. PKG-I is a cGMP-dependent protein kinase that mediates biological activity of NO via the NO/cGMP/PKG-I signaling pathway [21,26,27]. PKG-Iα and PKG-Iβ are alternative splices of a common gene product [28,29]. While sharing a common catalytic domain, PKG-Iα and PKG-Iβ are being activated by different concentrations of NO [30]. Low concentrations of NO (0.01–1 nM) activate PKG-Iα isoform which promotes cell survival and proliferation [27,31–37]. In contrast, higher concentrations of NO (1–50 nM) activate the PKG-Iβ isoform, which causes inhibition of cell proliferation [27,35]. PKG-I isoforms are critical to understanding the concentration-dependent biological effects of NO. However, the studies of PKG-I isoforms remain technically challenging due to the inability to resolve them on 1D gel electrophoresis and the cross reactivity between their antibodies. We show that cIEF immunoassay permits detection of PKG-I isoforms in various cell types. Most significantly, cIEF immunoassay permits simultaneous profiling of PKG-Iα, PKG-Iβ, Akt, ERK1, and ERK2 during fat cell differentiation.

## Materials and Methods

### Cell lines and tissues

The following cell lines were purchased from American Type Culture Collection (ATCC, Manassas, VA): HeLa (Cat. No. CCL-2), HUVEC (Cat. No. PCS-100-013), MCF-7 (Cat. No. HTB-22), NCI-H2052 (Cat. No. CRL-5915), and NG108-15 (Cat. No. HB-12317). Primary human omental preadipocytes were purchased from Zen-Bio (Cat. No. OP-F-3, Research Triangle Park, NC). Human pancreatic islets were purchased from Lonza (Cat. No. 201983, Allendale, NJ).

### Recombinant proteins

Recombinant PKG-I isoforms, PKG-Iβ (Cat. No. 14–650) and PKG-Iα (Cat. No. 14-688M) were purchased from Millipore (Billerica, MA).

### 1D Western blots

1D Western blots were performed using the following primary antibodies from Cell Signaling Techonology (Danvers, MA): ERK1/2 (Cat. No. 9107), PKG-I (Cat. No. 3248), eNOS (Cat. No. 9586), phospho-eNOS (Ser1177) (Cat. No. 9570) and from Abcam (Cambridge, MA): β-actin (Cat. No. ab8224). Secondary antibodies were purchased from LI-COR (Lincoln, Nebraska, Cat. No. 92668070). Immunoblots were detected with the Odyssey CLx imaging system (LI-COR).

### 2D Western blots

2D Western blots of recombinant PKG-I isoforms were performed by Kendrick Laboratories (Madison, WI). Recombinant protein was diluted to 0.0067 mg/ml in 1:1 diluted SDS boiling buffer: Urea sample buffer. A total of 1 μg of recombinant protein or 150 μl was loaded for isoeletric focusing. Carrier ampholine method of isoelectric focusing was carried out in a glass tube of inner diameter 3.3 mm using 2.0% pH 4–8 mix Servalytes (Serva, Heidelberg, Germany) for 20,000 volt-hrs. After equilibration for 10 minutes in 10% glycerol, 50 mM dithiothreitol, 2.3% SDS and 0.0625 M tris, pH 6.8, each tube gel was sealed to the top of a stacking gel that overlaid a 10% acrylamide slab gel. SDS slab gel electrophoresis was carried out for about 5 hours at 25 mA/gel. After slab gel electrophoresis, the gels were placed in transfer buffer (10 mM CAPS, pH 11.0, 10% MeoH) and transblotted onto a PVDF membrane overnight at 225 mA and approximately 100 volts/ two gels. The following proteins (Sigma Chemical Co., St. Louis, MO and EMD Millipore, Billerica, MA) were used as molecular mass standards: myosin (220,000), phosphorylase A (94,000), catalase (60,000), actin (43,000) carbonic anhydrase (29,000) and lysozyme (14,000). The blots were wet in 100% methanol, rinsed briefly in Tween -20 tris buffered saline (TTBS), and blocked for two hours in 5% Non Fat Dry Milk in TTBS. The blots were then incubated in primary antibody against PKG-I (Cat. No. 3248, Cell Signaling Technology) overnight and rinsed 3 x 10 minutes in TTBS. The blots were then placed in secondary antibody (Cat. No. NA931V, GE, Pittsburgh, PA) for two hours, rinsed in TTBS, treated with ECL, and exposed to X-ray film.

### Capillary isoelectric focusing immunoassays

cIEF systems purchased from ProteinSimple (NanoPro 100 & NanoPro 1000, Santa Clara, CA, USA) were employed for proteomics profiling. Samples of 400-nanoliter volume were separated by isoelectric focusing using the 12-capillary system (NanoPro 100) or the 96-capillary system (NanoPro 1000), followed by immobilization of the proteins onto the inner capillary walls. Subsequently, primary antibodies and secondary HRP-linked antibodies (Cat. No. 040656AB, Protein Simple) were introduced into the capillaries, followed by chemiluminescence detection reagents. For cell cultures, cells were first lysed with M-Per lysis buffer (Pierce, Rockford, Il, USA) in the presence of protease and phosphatase inhibitors. Total cell lysates were prepared in Premix G2 pH 5–8 separation gradient containing pI standards (ProteinSimple). For recombinant protein, recombinant protein was diluted in Bicine/CHAPS Lysis and Sample Diluent (Cat. No. 040–764, Protein Simple) which included DMSO inhibitor (Cat. No. 040–510, Protein Simple) and bovine serum albumin (0.5 mg/ml final concentration). Diluted protein was mixed with Premix G2 pH3-8 (Cat. No. 040–968, Protein Simple), pI standard Ladder 1 (Cat. No. 040–644), and pI standard 9.7 (Cat. No. 040–790) to 1:3 ratio. Separation time was 50 minutes at 15,000 MicroWatts. The incubation times were 110 and 55 minutes, respectively. Primary antibodies used were: PKG-I (Cat. No. 3248, Cell Signaling Technology), Akt1/2/3 (Cat. No. sc-8312, Santa Cruz Biotechnologies, Dallas, TX), and ERK1/2 (Cat. No. 06–182, Millipore All samples were loaded in triplicate into capillaries to evaluate consistency in capillary-to-capillary measurement. Each experiment was done in duplicate or triplicate to ensure repeatability. A minimum of 6 cIEF measurements were done for each sample. Representative data were presented in the figures. Unless specified, an average of 40 ng of total cellular protein was loaded into each capillary. Average exposure time during signal detection was 240 seconds.

### Fat cell differentiation

Primary human omental preadipocytes were grown to confluence in omental preadipocyte medium (Cat. No. OM-PM, Zen-Bio, Research Triangle Park, NC). On day 0, cells were cultured in omental differentiation medium (Cat. No. OM-DM, Zen-Bio). From day 8 to day 16, cells were cultured in omental adipocyte medium (Cat. No. OM-AM, Zen-Bio).

### Coherent anti-Stokes Raman scattering (CARS) microscopy

A home-built CARS microscope was employed to visualize lipid droplets in differentiating fat cell. The experimental setup of this microscope was described previously [38]. The vibrational frequency difference was tuned to 2851 cm^-1^, which matches the CH_2_ symmetric stretch vibration. Image was acquired at 1 second per frame and processed with NIH ImageJ.

## Results

**Fig 1:**
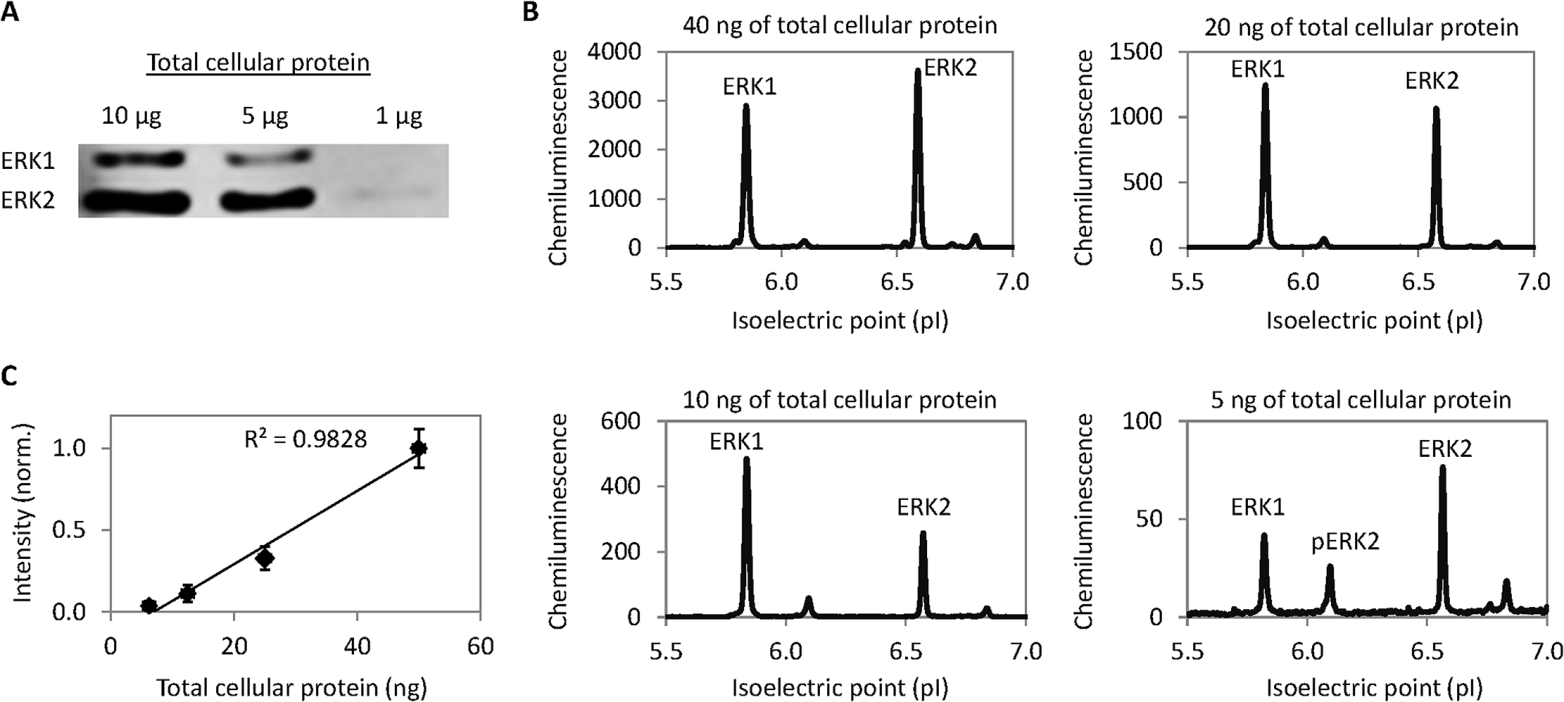
Detection sensitivity of 1D Western blots versus capillary isoelectric focusing immunoassays. (**A**) 1D Western blots with antibodies against ERK1 and ERK2 using micrograms of total cellular protein. (**B**) cIEF immunoassays with antibodies against ERK1 and ERK2 using nanograms for total cellular protein. (**C**) Integrated ERK1 and ERK2 chemiluminescent intensity as a function of total cellular protein described in (**B**). Error bars are standard deviation values of duplicate experiments.

The performance of cIEF immunoassay was compared to 1D Western blot for the detection of ERK1 and ERK2 in HELA total cell extracts. ERK1 and ERK2 could be reliably detected in 5 μg of total cellular protein with 1D Western blots (**Fig 1A**). The difference in the molecular mass of ERK1 (M.M.43 kD) and ERK2 (M.M. 41 kD) permitted their clear separation on 1D polyacrylamide gel electrophoresis. In contrast, ERK1 and ERK2 could be detected with 5 ng of total cellular protein with cIEF immunoassays (**Fig 1B**). The difference in isoelectric values of ERK1 and ERK2 permitted their clear separation with cIEF [20]. Integrated chemiluminescence signal intensity of ERK1, ERK2, and their phosphorylated forms was linearly correlated with the dilution of HELA total cellular protein (**Fig 1C**). It is unclear the reason for the variation between the ratio of ERK isoforms following dilution. However, similar observation has been reported previously for ERK isoforms in prostate LNCaP cells [20]. Variation between the ratio of protein and antibody concentration during dilution, where antibody concentration remained the same while protein concentration was continuously diluted, could be a potential source of error [19]. Nonetheless, in this specific demonstration, cIEF immunoassay could detect ERK1 and ERK2 in HELA total cell extracts using one thousand times less sample quantity compared to 1D WB.

**Fig 2:**
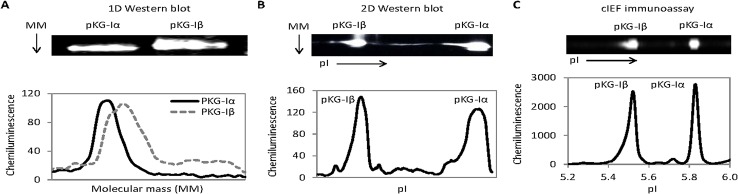
Detection of PKG-I isoforms using 1D and 2D Western blots and cIEF immunoassays. (**A**) 1D Western blot image (upper panel) and chemiluminescent intensity as a function of molecular mass plot (lower panel). (**B**) Truncated 2D Western blot images (upper panels) and chemiluminescent intensity as a function of isoelectric points plot (lower panel). (**C**) cIEF immunoassay images (upper panels) and chemiluminescent intensity as a function of isoelectric points plot (lower panel).

Isoelectric focusing (IEF) is a powerful means to separate proteins based on their electric charge differences. To highlight the capability of IEF, recombinant PKG-Iα (76.4 kD) and PKG-Iβ (77.8 kD) proteins were resolved with 1D WB, 2D WB, and cIEF immunoassay. On 1D WB, PKG-Iα and PKG-Iβ could not be separated from one another on the basis of molecular masss (**Fig 2A**). On 2D WB, recombinant PKG-Iα and PKG-Iβ could be clearly separated from one another on the basis of charge differences (**Fig 2B**). Similarly, cIEF immunoassay data concurred with 2D WB, where PKG-Iα and PKG-Iβ could be clearly resolved due to their differences in pI values (**Fig 2C**). However, it should be noted that 1 μg and 10 pg of recombinant PKG-I isoforms were used for 2D WB and cIEF immunoassay, respectively. This specific comparison demonstrated that cIEF immunoassay achieved the reliability of 2D WB for resolving recombinant PKG-Iα and PKG-Iβ using one hundred thousand times less sample quantity.

**Fig 3:**
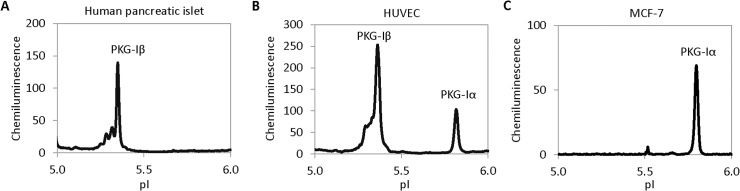
Differential expression of PKG-I isoforms in cell lines and tissues. (**A**) Human pancreatic islets exhibited only the presence of PKG-Iβ. (**B**) Human umbilical vascular endothelial cells (HUVEC) exhibited both PKG-Iα and PKG-Iβ. (**C**) Mammary cancer cells MCF-7 exhibited only PKG-Iα.

Taking advantage of the ability of cIEF to resolve PKG-I isoforms, the expression levels of PKG-Iα and PKG-Iβ were examined in various tissue types. In tissue extracts from human pancreatic islet, only the expression of PKG-Iβ was observed (**Fig 3A**). In cultured human umbilical vein endothelial cells (HUVEC), both PKG-Iα and PKG-Iβ were present, with PKG-Iβ being the dominant isoform (**Fig 3B**). In contrast, cultured MCF-7 breast cancer cell line exhibited only PKG-Iα isoform (**Fig 3C**). Differential expression of PKG-I isoforms in various cell types suggests differential sensitivity to the biological effects of NO.

**Fig 4:**
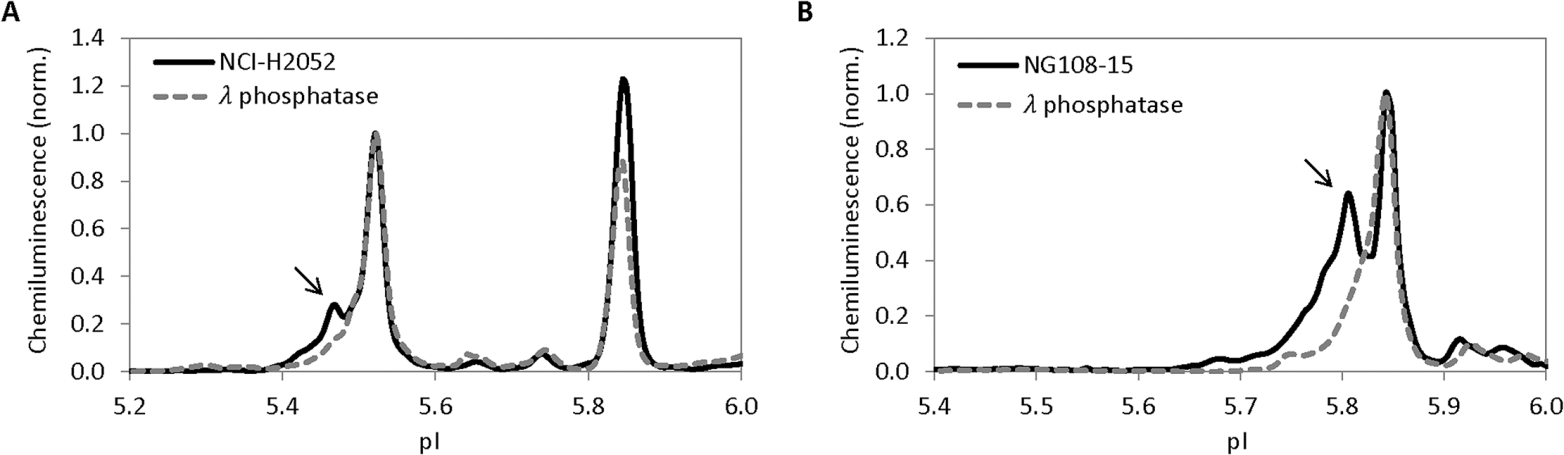
Detection of phosphorylation of PKG-Iα and PKG-Iβ with cIEF immunoassays. (**A**) Multiple PKG-Iβ peaks were detected in NCI-H2052 total cell lysates (solid line). Left-shifted PKG-Iβ peak was removed following treatment of NCI-H2052 total cell lysates with λ phosphatase (dashed line). Peak intensity was normalized to 1 for PKG-Iβ. (**B**) Multiple PKG-Iα peaks were detected in NG108-15 total cell lysates (solid line). Left-shifted PKG-Iα was removed following treatment of NG108-15 total cell lysates with λ phosphatase (dashed line). Peak intensity was normalized to 1 for PKG-Iβ and PKG-Iα. in **A** and **B**, respectively, to permit clear visualization of pI shift following λ phosphatase treatment.

cIEF immunoassay was also applied to detect phosphorylation of PKG-I isoforms. Using total cell lysates of cultured human metastatic lung cancer cell line NCI-H2052, multiple peaks for PKG-Iβ were observed (**Fig 4A**). Treatment of NCI-H2052 total cell lysate with λ-phosphatase removed the left-shifted peak which indicated that this peak might be associated with phosphorylated PKG-Iβ (**Fig 4A, arrow**). On the other hand, total cell lysates of cultured neuroblastoma somatic cell hybrid NG108-15 exhibited multiple peaks for PKG-Iα (**Fig 4B**). Treatment of NG108-15 total cell lysate with λ-phosphatase removed the left-shifted peak, which indicated that this peak might be associated with phosphorylated PKG-Iα (**Fig 4B, arrow**). Clearly, cIEF immunoassay provided a sensitive means to detect phosphorylation of PKG-Iα and PKG-Iβ.

**Fig 5:**
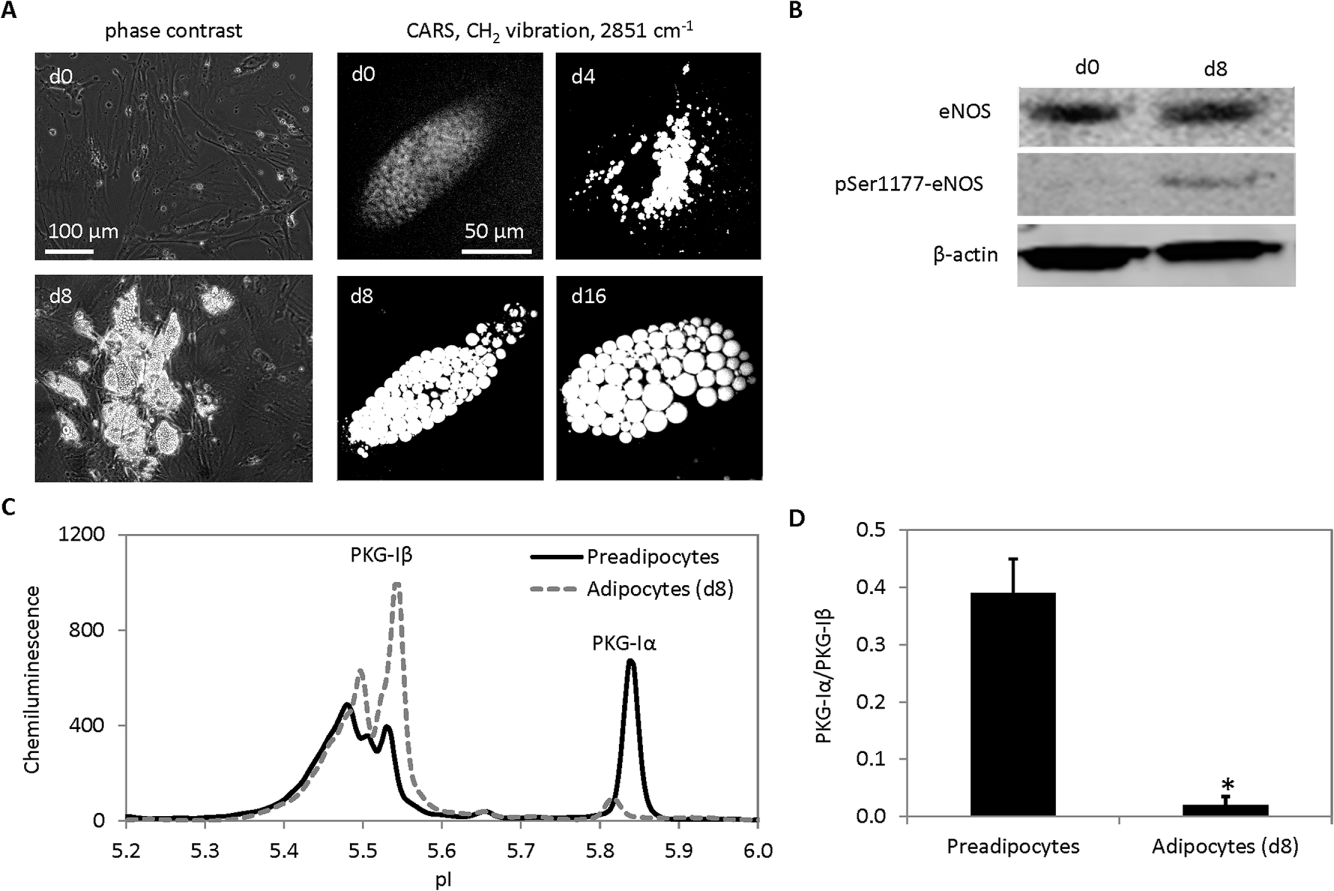
Differential expression of PKG-I isoforms during fat cell differentiation. (**A**) Images of human omental preadipocytes undergoing differentiation into adipocytes taken with phase contrast microscopy (left panels) and coherent anti-Stokes Raman scattering (CARS) microscopy (right panels). (**B**) Increased phosphorylation of endothelial nitric oxide synthase (eNOS) at Serine residue 1177 detected with 1D Western blots. (**C**) Expression of both PKG-Iα and PKG-Iβ isoforms in preadipocytes (solid line) and expression of only PKG-Iβ isoform in adipocytes (dashed line). (**D**) Normalized ratios of PKG-Iα/PKG-Iβ as a function of cell differentiation at day 0 (preadipocytes) and day 8 (adipocytes). Error bars are standard deviation values of triplicate measurements. Asterisk indicates p-value <0.01 versus preadipocytes.

Next, cIEF immunoassay was applied to monitor the expression of PKG-I isoforms during the differentiation of human omental preadipocytes into adipocytes. The NO/cGMP/PKG-I signaling pathway was known to play a role in fat cell differentiation [39–42]. It was shown that the production of NO increased in vascular endothelial cells as a consequence of insulin-stimulated phosphorylation of eNOS at Ser1177 by Akt [43]. During fat cell differentiation, adipocytes could be easily detected by the accumulation of lipid droplets that scattered light via phase contrast microscopy (**Fig 5A, left panels**) or by the accumulation of hydrocarbon lipid chains that produced strong CH_2_ molecular vibration via coherent anti-Stokes Raman scattering microscopy (**Fig 5A, right panels**) [44]. Increased phosphorylation of eNOS at Ser1177 was also observed during the differentiation of preadipocytes into adipocytes (**Fig 5B**). cIEF immunoassays revealed that preadipocytes expressed both isoforms of PKG-I (**Fig 5C**). Interestingly, the expression of PKG-Iα was strongly suppressed in mature adipocytes. Mature adipocytes expressed only PKG-Iβ and phosphorylated PKG-Iβ. Quantitatively, preadipocytes expressed both PKG-Iα and PKG-Iβ isoforms with the PKG-Iα/PKG-Iβ ratio of 0.39 (**Fig 5D**). In contrast, adipocytes expressed mostly PKG-Iβ with the PKG-Iα/PKG-Iβ ratio of 0.02. The expression of PKG-I in preadipocytes and adipocytes has been reported using Western blot assays [40,42,45]. However, differential expression of PKG-I isoforms in preadipocytes and adipocytes was reported in this study for the first time.

**Fig 6:**
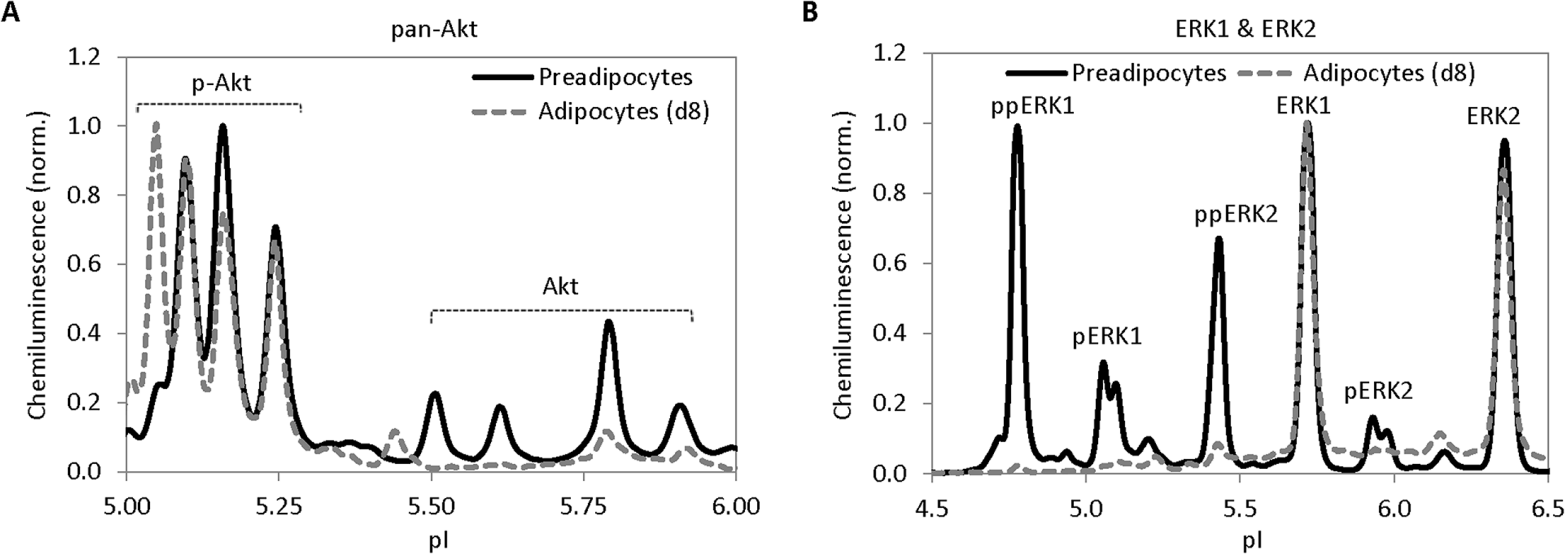
cIEF profiling of Akt, ERK1 and ERK2 during fat cell differentiation. (**A**) pan-Akt in preadipocytes (solid line) versus adipocytes (dashed line). (**B**) ERK 1 and ERK2 in preadipocytes (solid line) versus adipocytes (dashed line). Peak intensity was normalized to 1 in both **A** and **B** to permit clear visualization of the presence or absence of new peaks along pI axis.

In addition to profiling PKG-I isoforms, cIEF immunoassay was also employed to profile the phosphorylation of Akt and ERK isoforms during fat cell differentiation. Following the differentiation of preadipocytes into adipocytes, a distinctive Akt peak appeared at pI value of 5.05 in adipocytes that was absent in preadipocytes (**Fig 6A**). Assignment of pI values to different Akt isoforms or phosphorylated Akt isoforms was accomplished previously [46,47]. A left-shifted peak on a pI axis generally indicated addition of negatively charged molecules such as phosphate (PO_4_^3-^) groups onto the Akt protein structure. The appearance of the Akt peak at pI 5.05 was likely a consequence of increased Akt phosphorylation in adipocytes compared to preadipocytes. In contrast, the phosphorylation level of ERK1 and ERK2 decreased as a function of fat cell differentiation (**Fig 6B**). Peaks assignment of ERK1 and ERK2 and their phosphorylated isoform was accomplished previously by O’Neill *et al*. [20]. Using previously assigned peaks, it became obvious that phosphorylation isoforms of ERK1 and ERK2 including ppERK1, pERK1, ppERK2, and pERK2 were completely suppressed in adipocytes compared to preadipocytes. cIEF immunoassay proved to be a robust and versatile means for fat cell differentiation proteomics.

## Discussion

In this study, the capability of cIEF immunoassay for proteomic profiling was demonstrated. Compared to standard 1D and 2D Western blots for protein detection, cIEF immunoassay exhibited 3 to 5 orders of magnitude higher sensitivity, respectively. This capability permits reliable measurement of protein expression levels in several nanograms of tissue samples. In addition, cIEF immunoassay was sensitive to changes in pI values associated with protein phosphorylation. Taken together, cIEF immunoassay is a robust means for proteomic profiling using small sample quantity.

Profiling changes in protein PTM is critical to unraveling the mechanisms underlying fate commitment of mesenchymal stem cells into fat cells. Here, cIEF immunoassay was employed to highlight significant changes to three kinases of three different signaling pathways: PKG-I isoforms of the NO signaling pathway, Akt of the insulin signaling pathway, and ERK isoforms of the MAPK signaling pathway. We reported a novel observation on the suppression of PKG-Iα expression level during fat cell differentiation. Predominant expression of PKG-Iβ in mature adipocytes suggests a shift toward inhibition of cell proliferation for the NO signaling pathway [27]. Similarly, reduced phosphorylation level of ERK isoforms promotes cell growth arrest and fat cell differentiation [48]. On the other hand, increased phosphorylation of Akt supports increased insulin signaling activity that promotes the expression of adipocyte genes and lipogenesis [49]. Fat cell differentiation is a highly regulated process that requires participation of proteins of numerous signaling pathways [49]. High sample throughput of up to 96 samples per run of the cIEF immunoassay system provides an ideal platform for the analysis of interaction between complex signaling networks during cell fate determination.

The significance of the NO/cGMP/PKG-I signaling pathway in cardiovascular system has been well-demonstrated with vascular smooth muscle relaxation and platelet disaggregation [50]. Therapeutic intervention strategies that influence with the NO/cGMP/PKG-I signaling pathway to treat erectile dysfunction and pulmonary hypertension have been widely deployed [51]. In recent years, participation of the NO/cGMP/PKG-I signaling pathway in regulating non-vascular disease processes such as cancer and neurodegenerative diseases has also been reported [21,26,27,52]. It has been proposed that PKG-I isoforms mediate the concentration dependence of NO’s biological actions [27]. However, the difficulty in resolving PKG-I isoforms prevents the dissection of their distinctive roles. Interestingly, cIEF immunoassay permits clear detection of PKG-I isoforms. Differential expression levels of PKG-Iα and PKG-Iβ suggest different sensitivity to NO in different tissue types. Suppression of PKG-Iα expression during fat cell differentiation also indicates a differential sensitivity to NO in preadipocytes and adipocytes. The application of cIEF immunoassay to study the specific roles of PKG-I isoforms has the potential to selectively influence the NO/cGMP/PKG-I signaling pathway for therapeutic treatment of abnormalities of both vascular and non-vascular systems.
